# 5,6-Dimethyl-4-phenyl-2*H*-pyran-2-one

**DOI:** 10.1107/S1600536812011233

**Published:** 2012-03-21

**Authors:** Hai-Yun Xu, Sheng-Hai Guo, Kun Li, Xue-Sen Fan

**Affiliations:** a School of Chemistry and Environmental Science, Henan Key Laboratory for Environmental Pollution Control, Henan Normal University, Xinxiang, Henan 453007, People’s Republic of China

## Abstract

In the title compound, C_13_H_12_O_2_, the dihedral angle between the pyran­one and phenyl rings is 57.55 (9)°. In the crystal, the mol­ecules are linked by π–π stacking inter­actions between the parallel pyran­one rings of neighboring mol­ecules with distances of 3.5778 (11) Å and 3.3871 (11) Å between the planes. C—H⋯O interactions also occur.

## Related literature

For the bioactivity of 2*H*-pyran-2-ones, see: Puerta *et al.* (2005 ▶); Thaisrivongs *et al.* (1998 ▶); Appendino *et al.* (2007 ▶). For research on functionalized allenes, see: Fan *et al.* (2011 ▶); Zhang *et al.* (2011 ▶); Xu *et al.* (2012 ▶).
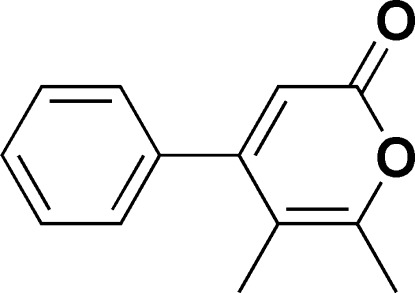


## Experimental

### 

#### Crystal data

C_13_H_12_O_2_
*M* *_r_* = 200.23
Monoclinic, 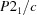

*a* = 7.654 (3) Å
*b* = 6.967 (3) Å
*c* = 20.629 (8) Å
β = 97.183 (4)°
*V* = 1091.4 (7) Å^3^
*Z* = 4
Mo *K*α radiation
μ = 0.08 mm^−1^
*T* = 296 K
0.39 × 0.37 × 0.28 mm

#### Data collection

Bruker SMART CCD area detector diffractometer
Absorption correction: multi-scan (*SADABS*; Bruker, 2007 ▶) *T*_min_ = 0.969, *T*_max_ = 0.978
7794 measured reflections
2032 independent reflections
1530 reflections with *I* > 2σ(*I*)
*R*_int_ = 0.021

#### Refinement

*R*[*F* ^2^ > 2σ(*F* ^2^)] = 0.045
*wR*(*F* ^2^) = 0.137
*S* = 1.04
2032 reflections
138 parameters
H-atom parameters constrained
Δρ_max_ = 0.18 e Å^−3^
Δρ_min_ = −0.15 e Å^−3^

### 

Data collection: *SMART* (Bruker, 2007 ▶); cell refinement: *SAINT* (Bruker, 2007 ▶); data reduction: *SAINT*; program(s) used to solve structure: *SHELXS97* (Sheldrick, 2008 ▶); program(s) used to refine structure: *SHELXL97* (Sheldrick, 2008 ▶); molecular graphics: *SHELXTL* (Sheldrick, 2008 ▶); software used to prepare material for publication: *SHELXTL*.

## Supplementary Material

Crystal structure: contains datablock(s) I, New_Global_Publ_Block. DOI: 10.1107/S1600536812011233/vm2163sup1.cif

Structure factors: contains datablock(s) I. DOI: 10.1107/S1600536812011233/vm2163Isup2.hkl

Supplementary material file. DOI: 10.1107/S1600536812011233/vm2163Isup3.cdx

Supplementary material file. DOI: 10.1107/S1600536812011233/vm2163Isup4.cdx

Additional supplementary materials:  crystallographic information; 3D view; checkCIF report

## Figures and Tables

**Table 1:** Hydrogen-bond geometry (Å, °)

*D*—H⋯*A*	*D*—H	H⋯*A*	*D*⋯*A*	*D*—H⋯*A*
C8—H8⋯O2^i^	0.93	2.53	3.384 (2)	152
C13—H13*A*⋯O2^ii^	0.96	2.47	3.372 (3)	156

Symmetry codes: (i) 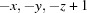; (ii) 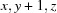.

**Fig. 1.**
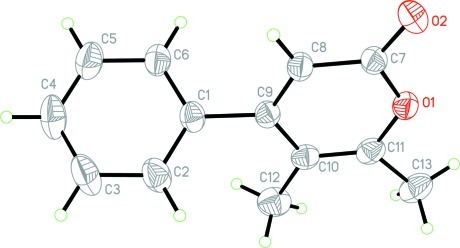
Molecular structure of the title compound, with displacement ellipsoids drawn at the 30% probability level.

**Fig. 2.**
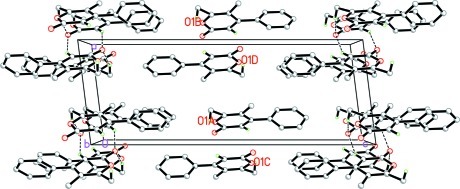
Crystal packing of the title compound, viewed along the b axis. Intermolecular C—H···O hydrogen bonds are shown as dashed lines, only H atoms involved in hydrogen bonds are shown. π-π stacking interactions between the parallel pyranone rings of neighboring molecules are observed.

## supplementary crystallographic information

### Comment

2*H*-Pyran-2-one derivatives are highly desirable synthetic targets since they are known to have antimicrobial, antineoplastic, and anti-HIV effects (Puerta *et al.*, 2005; Thaisrivongs *et al.*, 1998; Appendino *et al.*, 2007). During our search for new synthetic methodologies by taking the advantages of the versatile reactivity of functionalized allenes (Fan *et al.*, 2011; Zhang *et al.*, 2011), we developed a novel protocol for the preparation of 2*H*-pyran-2-ones through an acid-catalyzed domino reaction of 3-hydroxyhexa-4,5-dienoates (Xu *et al.*, 2012). Herein, we would like to report the structure of one of the products we obtained.

In the title compound (Fig. 1), all the bond lengths and bond angles are within normal ranges. All the atoms connected with the pyranone ring are in the pyranone plane with a maximal deviation of 0.052 (2) Å for substituent C12. The dihedral angle between the pyranone ring and the phenyl ring is 57.55 (9)°.

In the crystal structure, the molecules are connected *via* intermolecular C—H···O hydrogen bonds (Table 1, Fig. 2). The neighboring O1B-pyranone ring, O1D-pyranone ring, O1A-pyranone ring and O1C-pyranone ring [symmetry code: (B) 1 + *x*, *y*, *z*; (C) -*x*, 1 - *y*, 1 - *z*; (D) 1 - *x*, 1 - *y*, 1 - *z*] are parallel with the distance between the O1D ring and O1A ring being 3.5778 (11) Å and the distance between the O1A ring and O1C ring being 3.3871 (11) Å. The short face-to-face separation clearly indicates the existence of π-π stacking between the pyranone rings.

### Experimental

To a flask containing methyl 3-hydroxy-4-methyl-3-phenylhexa-4,5-dienoate (1 mmol) were added CH_2_Cl_2_ (5 ml) and conc. H_2_SO_4_ (0.1 mmol). The solution was stirred at room temperature until completion as monitored by TLC. The reaction was quenched with aqueous NaHCO_3_, and then extracted with ethyl acetate (5 ml × 3). The combined organic phases were dried, filtered and concentrated under vacuum. The residue was purified by column chromatography on silica gel eluenting with petroleum ether-ethyl acetate (10:1 *v*/*v*) to give the title compound as colorless solids with a yield of 90%. Single crystals, suitable for X-ray diffraction analysis, were obtained by slow evaporation of solvent from a petroleum ether-dichloromethane (3:1 *v*/*v*) solution.

### Refinement

The H atoms were included at calculated positions and were refined as riding atoms: C—H = 0.93 and 0.96 Å for aromatic and methyl H atoms, respectively, with *U*_iso_(H) =*x*×*U*_eq_ (C), where *x* = 1.5 for methyl H, and *x* = 1.2 for aromatic H atoms.

### Crystal data

**Table:**

C_13_H_12_O_2_	*F*(000) = 424
*M**_r_* = 200.23	*D*_x_ = 1.219 Mg m^−^^3^
Monoclinic, *P*2_1_/*c*	Mo *K*α radiation, λ = 0.71073 Å
Hall symbol: -P 2ybc	Cell parameters from 2249 reflections
*a* = 7.654 (3) Å	θ = 2.7–25.9°
*b* = 6.967 (3) Å	µ = 0.08 mm^−^^1^
*c* = 20.629 (8) Å	*T* = 296 K
β = 97.183 (4)°	Block, colourless
*V* = 1091.4 (7) Å^3^	0.39 × 0.37 × 0.28 mm
*Z* = 4	

### Data collection

**Table:**

Bruker SMART CCD area detector diffractometer	2032 independent reflections
Radiation source: fine-focus sealed tube	1530 reflections with *I* > 2σ(*I*)
Graphite monochromator	*R*_int_ = 0.021
phi and ω scans	θ_max_ = 25.5°, θ_min_ = 2.7°
Absorption correction: multi-scan (*SADABS*; Bruker, 2007)	*h* = −9→9
*T*_min_ = 0.969, *T*_max_ = 0.978	*k* = −8→8
7794 measured reflections	*l* = −24→24

### Refinement

**Table:**

Refinement on *F*^2^	Primary atom site location: structure-invariant direct methods
Least-squares matrix: full	Secondary atom site location: difference Fourier map
*R*[*F*^2^ > 2σ(*F*^2^)] = 0.045	Hydrogen site location: inferred from neighbouring sites
*wR*(*F*^2^) = 0.137	H-atom parameters constrained
*S* = 1.04	*w* = 1/[σ^2^(*F*_o_^2^) + (0.0659*P*)^2^ + 0.2202*P*] where *P* = (*F*_o_^2^ + 2*F*_c_^2^)/3
2032 reflections	(Δ/σ)_max_ = 0.001
138 parameters	Δρ_max_ = 0.18 e Å^−^^3^
0 restraints	Δρ_min_ = −0.15 e Å^−^^3^

### Special details

**Table:**

Geometry. All e.s.d.'s (except the e.s.d. in the dihedral angle between two l.s. planes)are estimated using the full covariance matrix. The cell e.s.d.'s are takeninto account individually in the estimation of e.s.d.'s in distances, anglesand torsion angles; correlations between e.s.d.'s in cell parameters are onlyused when they are defined by crystal symmetry. An approximate (isotropic)treatment of cell e.s.d.'s is used for estimating e.s.d.'s involving l.s. planes.
Refinement. Refinement of *F*^2^ against ALL reflections. The weighted *R*-factor *wR* andgoodness of fit *S* are based on *F*^2^, conventional *R*-factors *R* are basedon *F*, with *F* set to zero for negative *F*^2^. The threshold expression of*F*^2^ > σ(*F*^2^) is used only for calculating *R*-factors(gt) *etc*. and isnot relevant to the choice of reflections for refinement. *R*-factors basedon *F*^2^ are statistically about twice as large as those based on *F*, and *R*-factors based on ALL data will be even larger.

### Fractional atomic coordinates and isotropic or equivalent isotropic displacement parameters (Å^2^)

**Table:**

	*x*	*y*	*z*	*U*_iso_*/*U*_eq_	
C1	0.2300 (2)	0.4016 (3)	0.64903 (8)	0.0547 (4)	
C2	0.1478 (3)	0.5353 (3)	0.68457 (9)	0.0749 (6)	
H2	0.0883	0.6385	0.6634	0.090*	
C3	0.1541 (3)	0.5154 (4)	0.75160 (10)	0.0907 (8)	
H3	0.0979	0.6049	0.7753	0.109*	
C4	0.2425 (3)	0.3647 (4)	0.78333 (10)	0.0894 (7)	
H4	0.2464	0.3523	0.8284	0.107*	
C5	0.3249 (3)	0.2329 (4)	0.74867 (10)	0.0831 (7)	
H5	0.3861	0.1315	0.7703	0.100*	
C6	0.3180 (3)	0.2493 (3)	0.68175 (9)	0.0651 (5)	
H6	0.3727	0.1575	0.6585	0.078*	
C7	0.1397 (2)	0.2616 (2)	0.47092 (8)	0.0506 (4)	
C8	0.1532 (2)	0.2625 (2)	0.54020 (8)	0.0508 (4)	
H8	0.1145	0.1558	0.5615	0.061*	
C9	0.22072 (19)	0.4138 (2)	0.57639 (7)	0.0483 (4)	
C10	0.2838 (2)	0.5790 (2)	0.54428 (8)	0.0520 (4)	
C11	0.2706 (2)	0.5760 (2)	0.47847 (9)	0.0553 (4)	
C12	0.3667 (3)	0.7471 (3)	0.58167 (11)	0.0771 (6)	
H12A	0.4441	0.8121	0.5558	0.116*	
H12B	0.4326	0.7029	0.6215	0.116*	
H12C	0.2764	0.8339	0.5917	0.116*	
C13	0.3217 (3)	0.7292 (3)	0.43413 (11)	0.0775 (6)	
H13A	0.2281	0.8212	0.4264	0.116*	
H13B	0.3434	0.6731	0.3934	0.116*	
H13C	0.4265	0.7920	0.4540	0.116*	
O1	0.20108 (14)	0.42248 (16)	0.44270 (5)	0.0546 (3)	
O2	0.08210 (18)	0.13534 (19)	0.43387 (6)	0.0699 (4)	

### Atomic displacement parameters (Å^2^)

**Table:**

	*U*^11^	*U*^22^	*U*^33^	*U*^12^	*U*^13^	*U*^23^
C1	0.0496 (9)	0.0703 (11)	0.0427 (9)	0.0008 (8)	0.0004 (7)	−0.0027 (8)
C2	0.0710 (12)	0.0994 (15)	0.0514 (10)	0.0218 (11)	−0.0034 (9)	−0.0120 (10)
C3	0.0787 (14)	0.141 (2)	0.0513 (11)	0.0202 (14)	0.0051 (10)	−0.0242 (13)
C4	0.0815 (14)	0.143 (2)	0.0420 (10)	−0.0013 (15)	0.0022 (10)	0.0023 (13)
C5	0.0916 (15)	0.1017 (17)	0.0535 (12)	0.0055 (13)	−0.0009 (11)	0.0162 (11)
C6	0.0736 (12)	0.0735 (12)	0.0476 (10)	0.0039 (9)	0.0052 (8)	0.0042 (9)
C7	0.0523 (9)	0.0550 (10)	0.0451 (9)	0.0090 (7)	0.0079 (7)	0.0006 (8)
C8	0.0529 (9)	0.0560 (10)	0.0439 (9)	0.0036 (7)	0.0078 (7)	0.0037 (7)
C9	0.0434 (8)	0.0565 (9)	0.0443 (9)	0.0069 (7)	0.0020 (6)	0.0004 (7)
C10	0.0449 (9)	0.0502 (9)	0.0600 (10)	0.0062 (7)	0.0025 (7)	0.0014 (8)
C11	0.0469 (9)	0.0566 (10)	0.0640 (11)	0.0104 (8)	0.0129 (8)	0.0110 (8)
C12	0.0726 (13)	0.0640 (12)	0.0910 (15)	−0.0060 (10)	−0.0045 (11)	−0.0075 (10)
C13	0.0767 (13)	0.0691 (12)	0.0903 (15)	0.0091 (10)	0.0243 (11)	0.0282 (11)
O1	0.0615 (7)	0.0583 (7)	0.0452 (6)	0.0088 (6)	0.0109 (5)	0.0054 (5)
O2	0.0899 (10)	0.0664 (8)	0.0532 (7)	−0.0013 (7)	0.0083 (7)	−0.0133 (6)

### Geometric parameters (Å, º)

**Table:**

C1—C2	1.384 (3)	C7—C8	1.420 (2)
C1—C6	1.387 (2)	C8—C9	1.356 (2)
C1—C9	1.494 (2)	C8—H8	0.9300
C2—C3	1.384 (3)	C9—C10	1.440 (2)
C2—H2	0.9300	C10—C11	1.349 (2)
C3—C4	1.370 (3)	C10—C12	1.499 (2)
C3—H3	0.9300	C11—O1	1.369 (2)
C4—C5	1.366 (3)	C11—C13	1.489 (2)
C4—H4	0.9300	C12—H12A	0.9600
C5—C6	1.380 (3)	C12—H12B	0.9600
C5—H5	0.9300	C12—H12C	0.9600
C6—H6	0.9300	C13—H13A	0.9600
C7—O2	1.212 (2)	C13—H13B	0.9600
C7—O1	1.373 (2)	C13—H13C	0.9600
			
C2—C1—C6	118.86 (16)	C7—C8—H8	118.9
C2—C1—C9	121.69 (16)	C8—C9—C10	119.63 (15)
C6—C1—C9	119.41 (15)	C8—C9—C1	118.35 (15)
C1—C2—C3	120.0 (2)	C10—C9—C1	122.01 (15)
C1—C2—H2	120.0	C11—C10—C9	117.62 (15)
C3—C2—H2	120.0	C11—C10—C12	120.21 (17)
C4—C3—C2	120.5 (2)	C9—C10—C12	122.14 (16)
C4—C3—H3	119.7	C10—C11—O1	121.97 (15)
C2—C3—H3	119.7	C10—C11—C13	127.95 (18)
C5—C4—C3	119.86 (19)	O1—C11—C13	110.07 (16)
C5—C4—H4	120.1	C10—C12—H12A	109.5
C3—C4—H4	120.1	C10—C12—H12B	109.5
C4—C5—C6	120.4 (2)	H12A—C12—H12B	109.5
C4—C5—H5	119.8	C10—C12—H12C	109.5
C6—C5—H5	119.8	H12A—C12—H12C	109.5
C5—C6—C1	120.39 (19)	H12B—C12—H12C	109.5
C5—C6—H6	119.8	C11—C13—H13A	109.5
C1—C6—H6	119.8	C11—C13—H13B	109.5
O2—C7—O1	116.24 (15)	H13A—C13—H13B	109.5
O2—C7—C8	127.79 (16)	C11—C13—H13C	109.5
O1—C7—C8	115.97 (14)	H13A—C13—H13C	109.5
C9—C8—C7	122.13 (15)	H13B—C13—H13C	109.5
C9—C8—H8	118.9	C11—O1—C7	122.68 (13)
			
C6—C1—C2—C3	−0.1 (3)	C2—C1—C9—C10	59.0 (2)
C9—C1—C2—C3	177.71 (19)	C6—C1—C9—C10	−123.23 (18)
C1—C2—C3—C4	0.5 (4)	C8—C9—C10—C11	0.7 (2)
C2—C3—C4—C5	−0.1 (4)	C1—C9—C10—C11	179.62 (14)
C3—C4—C5—C6	−0.7 (4)	C8—C9—C10—C12	−177.38 (15)
C4—C5—C6—C1	1.2 (3)	C1—C9—C10—C12	1.5 (2)
C2—C1—C6—C5	−0.8 (3)	C9—C10—C11—O1	−0.3 (2)
C9—C1—C6—C5	−178.59 (17)	C12—C10—C11—O1	177.82 (15)
O2—C7—C8—C9	179.99 (16)	C9—C10—C11—C13	178.38 (16)
O1—C7—C8—C9	0.7 (2)	C12—C10—C11—C13	−3.5 (3)
C7—C8—C9—C10	−1.0 (2)	C10—C11—O1—C7	0.1 (2)
C7—C8—C9—C1	−179.88 (14)	C13—C11—O1—C7	−178.77 (14)
C2—C1—C9—C8	−122.10 (19)	O2—C7—O1—C11	−179.66 (14)
C6—C1—C9—C8	55.7 (2)	C8—C7—O1—C11	−0.3 (2)

### Hydrogen-bond geometry (Å, º)

**Table:**

*D*—H···*A*	*D*—H	H···*A*	*D*···*A*	*D*—H···*A*
C8—H8···O2^i^	0.93	2.53	3.384 (2)	152
C13—H13*A*···O2^ii^	0.96	2.47	3.372 (3)	156

Symmetry codes: (i) −*x*, −*y*, −*z*+1; (ii) *x*, *y*+1, *z*.

## References

[bb1] Appendino, G., Ottino, M., Marquez, N., Bianchi, F., Giana, A., Ballero, M., Sterner, O., Fiebich, B. L. & Munoz, E. (2007). *J. Nat. Prod.* **70**, 608–612. 10.1021/np060581r 17315926

[bb2] Bruker (2007). *SADABS*, *SMART* and *SAINT* Bruker AXS Inc., Madison, Wisconsin, USA.

[bb3] Fan, X., Wang, Y., Qu, Y., Xu, H., He, Y., Zhang, X. & Wang, J. (2011). *J. Org. Chem.* **76**, 982–985. 10.1021/jo102131y 21214220

[bb4] Puerta, D. T., Mongan, J., Tran, B. L., McCammon, J. A. & Cohen, S. M. (2005). *J. Am. Chem. Soc.* **127**, 14148–14149. 10.1021/ja054558o 16218585

[bb5] Sheldrick, G. M. (2008). *Acta Cryst.* A**64**, 112–122. 10.1107/S0108767307043930 18156677

[bb6] Thaisrivongs, S., Janakiraman, M. N., Chong, K.-T., Tomich, P. K., Dolak, L. A., Turner, S. R., Strohbach, J. W., Lynn, J. C., Horng, M.-M., Hinshaw, R. R. & Watenpaugh, K. D. (1998). *J. Med. Chem.* **39**, 2400–2410. 10.1021/jm950888f 8691434

[bb7] Xu, H., Zhang, X., He, Y., Guo, S. & Fan, X. (2012). *Chem. Commun.* **48**, 3121–3123. 10.1039/c2cc30247k 22343780

[bb8] Zhang, X., Jia, X., Fang, L., Liu, N., Wang, J. & Fan, X. (2011). *Org. Lett.* **13**, 5024–5027. 10.1021/ol201789z 21877737

